# Improvements in Physical Function and Pain Interference and Changes in Mental Health Among Patients Seeking Musculoskeletal Care

**DOI:** 10.1001/jamanetworkopen.2023.20520

**Published:** 2023-06-28

**Authors:** Wei Zhang, Som P. Singh, Amdiel Clement, Ryan P. Calfee, Janine D. Bijsterbosch, Abby L. Cheng

**Affiliations:** 1 Mallinckrodt Institute of Radiology, Washington University School of Medicine, St Louis, Missouri; 2 University of Missouri–Kansas City School of Medicine, Kansas City; 3 Washington University School of Medicine, St Louis, Missouri; 4 Division of Hand and Wrist, Department of Orthopaedic Surgery, Washington University School of Medicine, St Louis, Missouri; 5 Division of Physical Medicine and Rehabilitation, Department of Orthopaedic Surgery, Washington University School of Medicine, St Louis, Missouri

## Abstract

**Importance:**

Among patients seeking care for musculoskeletal conditions, there is mixed evidence regarding whether traditional, structure-based care is associated with improvement in patients’ mental health.

**Objective:**

To determine whether improvements in physical function and pain interference are associated with meaningful improvements in anxiety and depression symptoms among patients seeking musculoskeletal care.

**Design, Setting, and Participants:**

This cohort study included adult patients treated by an orthopedic department of a tertiary care US academic medical center from June 22, 2015, to February 9, 2022. Eligible participants presented between 4 and 6 times during the study period for 1 or more musculoskeletal conditions and completed Patient-Reported Outcomes Measurement Information System (PROMIS) measures as standard care at each visit.

**Exposure:**

PROMIS Physical Function and Pain Interference scores.

**Main Outcomes and Measures:**

Linear mixed effects models were used to determine whether improvements in PROMIS Anxiety and PROMIS Depression scores were associated with improved PROMIS Physical Function or Pain Interference scores after controlling for age, gender, race, and PROMIS Depression (for the anxiety model) or PROMIS Anxiety (for the depression model). Clinically meaningful improvement was defined as 3.0 points or more for PROMIS Anxiety and 3.2 points or more for PROMIS Depression.

**Results:**

Among 11 236 patients (mean [SD] age, 57 [16] years), 7218 (64.2%) were women; 120 (1.1%) were Asian, 1288 (11.5%) were Black, and 9706 (86.4%) were White. Improvements in physical function (β = −0.14; 95% CI, −0.15 to −0.13; *P* < .001) and pain interference (β = 0.26; 95% CI, 0.25 to 0.26; *P* < .001) were each associated with improved anxiety symptoms. To reach a clinically meaningful improvement in anxiety symptoms, an improvement of 21 PROMIS points or more (95% CI, 20-23 points) on Physical Function or 12 points or more (95% CI, 12-12 points) on Pain Interference would be required. Improvements in physical function (β = −0.05; 95% CI, −0.06 to −0.04; *P* < .001) and pain interference (β = 0.04; 95% CI, 0.04 to 0.05; *P* < .001) were not associated with meaningfully improved depression symptoms.

**Conclusions and Relevance:**

In this cohort study, substantial improvements in physical function and pain interference were required for association with any clinically meaningful improvement in anxiety symptoms, and were not associated with any meaningful improvement in depression symptoms. Patients seeking musculoskeletal care clinicians providing treatment cannot assume that addressing physical health will result in improved symptoms of depression or potentially even sufficiently improved symptoms of anxiety.

## Key Points

**Question:**

Among patients seeking musculoskeletal care, are improvements in physical function and pain interference associated with meaningful changes in symptoms of anxiety and depression?

**Findings:**

In this cohort study of 11 236 US adults seeking musculoskeletal care, improvement by 2.3 population-level standard deviations (SD) or more on PROMIS Physical Function or 1.2 SD or more on PROMIS Pain Interference were required for any association with meaningful improvement in anxiety symptoms. Improvements in physical function and pain interference were not associated with meaningfully improved depression symptoms.

**Meaning:**

These results suggest that clinicians and patients cannot assume that exclusively addressing the physical aspect of a musculoskeletal condition will improve symptoms of depression or potentially even anxiety.

## Teaser

This cohort study of US adults seeking musculoskeletal care examines whether self-reported improvements in physical function and pain were associated with improvements in anxiety and depression symptoms.

## Introduction

Physical and mental health have a complex bidirectional relationship. There is a high prevalence of comorbid physical and mental health impairment from conditions such as musculoskeletal disorders, anxiety, and depression, and patients seeking musculoskeletal care who have coexisting symptoms of anxiety and depression report more physical limitations and worse pain interference (ie, “consequences of pain on relevant aspects of a person’s life...including hindered engagement with social, cognitive, emotional, physical, and recreational activities”^1^) than those who do not.^2,3,4,5,6,7,8,9,10,11,12,13^

Mental health treatment for anxiety and depression can secondarily improve patients’ physical function and reduce their pain-related limitations, but the reverse phenomenon has not been as well investigated.^14,15,16,17,18,19^ That is, among patients seeking care for musculoskeletal conditions, there is mixed evidence regarding whether treatment of physical conditions is associated with spontaneous improvement in mental health symptoms.^20,21,22^ Currently, musculoskeletal clinicians and patients often focus on the treatment of physical concerns, with the hope that mental health–related symptoms will naturally improve as physical health improves.^23,24^ This practice may in part be related to unique barriers to accessing mental health care, such as societal stigma regarding mental illness, a lack of financial accessibility to mental health care, and a global shortage of mental health professionals.^25,26,27^ Furthermore, the structure of medical training is such that clinicians who subspecialize in treating physical impairments do not routinely receive training in addressing the mental health–related contributors to and sequelae of physical and pain-related impairments.^24,28^

Musculoskeletal clinicians also have discrepant opinions regarding whether addressing patients’ mental health falls within their professional role.^24,28,29^ Some musculoskeletal clinicians are interested in additional resources to better address mental health within the musculoskeletal care setting,^30^ but acquisition of these resources has remained challenging without widespread agreement regarding the need for this investment.^28,29,31^ A better understanding of the associations between physical health and mental health changes can guide musculoskeletal clinicians in: (1) the importance they place on addressing mental health–related symptoms as a component of their patient care, and (2) how they counsel patients regarding expectations of their symptom trajectory as their physical impairment is addressed.

The goal of this study was to determine whether, among patients seeking musculoskeletal care, self-reported improvements in physical function and pain interference are each associated with meaningful improvements in self-reported symptoms of anxiety and depression. We hypothesized that clinically meaningful improvement in physical function and pain interference would each be associated with significantly improved symptoms of anxiety and depression.

## Methods

This retrospective cohort study included patients who presented to a tertiary academic medical center between June 22, 2015, and February 9, 2022. All study data were extracted from the electronic medical record. Institutional review board approval was granted by Washington University in St Louis with a waiver of informed consent because this was a retrospective study of existing data. Study reporting follows the Strengthening the Reporting of Observational Studies in Epidemiology (STROBE) reporting guidelines for observational studies.

### Participants

All study participants were adults aged 18 years or older who sought evaluation and management of 1 or more musculoskeletal conditions at an outpatient clinic of the study institution’s orthopedic department. Prior to each clinic evaluation all patients completed Patient-Reported Outcomes Measurement Information System (PROMIS) Computer Adaptive Test (CAT) Anxiety version 1.0, Depression version 1.0, Physical Function version 1.2, and Pain Interference version 1.1 measures as part of standard clinical care. Patient visits were excluded from consideration if they were missing scores for any of these measures. To capture time-varying associations between these PROMIS variables of interest while also maximizing statistical power, our primary cohort included a consecutive sample of patients who had between 4 and 6 eligible clinic visits during the study period (eMethods in the Supplement 1).

### Exposure and Outcome Measures

Our exposures of interest were patients’ level of physical function and pain interference over time, which were quantified using their PROMIS CAT Physical Function version 1.2 and PROMIS CAT Pain Interference version 1.1 scores from each clinic visit, respectively.^8,32,33,34^ Our outcomes of interest were patients’ symptoms of anxiety and depression, which were quantified using their PROMIS CAT Anxiety version 1.0 and Depression version 1.0 scores from each clinic visit, respectively.^35,36^

PROMIS is a set of self-reported measures that was developed by the National Institutes of Health to measure multiple domains of health, irrespective of a person’s underlying medical conditions.^37^ Scores for each PROMIS measure are normalized to a representative sample of the general US population, with a mean score of 50 and standard deviation of 10. A higher score represents more of the domain being assessed, such that a high score on PROMIS Physical Function is favorable, whereas a low score on PROMIS Pain Interference, Anxiety, and Depression is favorable. Clinically meaningful effect sizes for symptom improvement were defined as at least 3.0 points for PROMIS Anxiety and 3.2 points for PROMIS Depression, which, based on previous studies, are the minimum clinically important differences among patients with musculoskeletal pain that also exceed the standard error of measurement for each PROMIS CAT at the study institution.^38,39^

### Confounding Variables

Patients’ age, self-reported gender, and self-reported race (based on categories defined by the US Census Bureau) were also available in the medical record and were included in all statistical models to account for potential influences of confounders. Race was included as a variable because it has previously been associated with self-reported physical and mental health in some contexts.

### Generalizability Analyses

A second cohort was analyzed to assess the generalizability of our findings to patients who had relatively fewer visits to the study institution’s orthopedic department. Potential reasons for fewer visits include, but were not limited to, musculoskeletal conditions that were less chronic or more responsive to initial treatment, conditions that were less severe or complex, conditions that warranted referral for alternative treatment, and social factors such as patient transportation barriers. This generalizability cohort consisted of a consecutive sample of patients who had only 3 clinic visits during the 6-year study period (as compared with our primary cohort, which had 4 to 6 visits). These patients were not included in our primary cohort because the smaller number of visits is not ideal to capture time-varying associations between the measures of interest.

### Statistical Analysis

Linear mixed effects models (LMM) were used for all analyses. LMM is a widely used statistical approach that incorporates fixed effects (ie, nonvarying coefficients of explanatory variables across all patients) and random effects (ie, varying coefficients within each patient) to estimate variance in longitudinal data with a multilevel or hierarchical structure. Here, we used LMM to estimate random effects of the grouping factors *patient* and *visit* (both as categorical variables) to account for individual-level variance (eg, different between-visit time intervals across patients) and assessment-level variance (eg, varying injury characteristics associated with the total number of clinic visits during the study period), respectively. Patients’ data from all clinic visits (ie, up to 6 visits for the primary cohort) were used for estimation. See eMethods in Supplement 1 for additional model specifications.

Separate models were run for PROMIS Anxiety and PROMIS Depression as the dependent variable (outcome measure) and for physical function and pain interference as the independent variable (exposure measure). First, we tested for a main effect of PROMIS Physical Function on PROMIS Anxiety and then on PROMIS Depression, with adjustment for confounding variables including age, gender, and race (categorized as White vs non-White because the sample was predominantly White). Because anxiety and depression symptoms are frequently comorbid and highly correlated,^40^ we also adjusted for PROMIS Depression and PROMIS Anxiety scores as covariates in the respective models in order to account for shared variance and to test for the main effect of physical function specific to either disorder. Calculated β coefficients below 0.06 were considered to indicate nonsignificance because they would represent changes in PROMIS Anxiety or Depression scores that would not reach our predefined minimum clinically meaningful thresholds, even if the greatest possible score change in PROMIS Physical Function or Pain Interference was achieved.

Next, to focus on patients who, according to our hypothesis, would be most likely to achieve clinically meaningful improvement in symptoms of anxiety and depression, we repeated all analyses on the subgroup of patients whose physical function meaningfully improved (ie, 5-point score increase in PROMIS Physical Function between their first and last clinic visit) during the study period (eFigure in Supplement 1). While the precise threshold for meaningful improvement varies based on the patient population and is not universally agreed upon, 5 points was chosen as the cutoff value for this study because it corresponds to a moderate effect size change (ie, 0.5 standard deviations) and has repeatedly been found to represent a clinically important difference across various orthopedic patient populations.^38,41,42^

We then repeated all statistical procedures using PROMIS Pain Interference as the exposure measure, rather than PROMIS Physical Function. Separate models were run for each of these 2 measures without adjusting for their shared variance because: (1) although physical function and pain interference are closely related, they are conceptually different constructs, and (2) the purpose of this study was to evaluate for longitudinal associations between these physical health constructs and mental health symptoms, but not necessarily to identify the specific influence of each construct on mental health symptoms. Meaningfully improved pain interference was defined as a 5-point decrease in PROMIS Pain Interference.^38,43,44^

All statistical procedures were also repeated in follow-up tests using the generalizability cohort of patients who only had 3 clinic visits during the study period (as compared with our primary cohort which had 4 to 6 visits). The sample size of each cohort was determined by the availability of eligible patients. Given the large available sample size, the relatively small proportion of patients with missing PROMIS scores were excluded from analysis based on the study eligibility criteria. *P* values were derived from F tests using Satterthwaite methods.^45^ To account for multiple comparisons, false discovery rate (FDR) corrections were applied to all the models using the Benjamini-Hochberg approach.^46^ Hypothesis testing was 2-sided, and a *P* value < .05 after FDR corrections was considered statistically significant. All statistical analyses were conducted using R version 4.10 (R Project for Statistical Computing). Linear mixed effects models were run using the lmerTest package.

## Results

### Demographics

**Figure 1.**
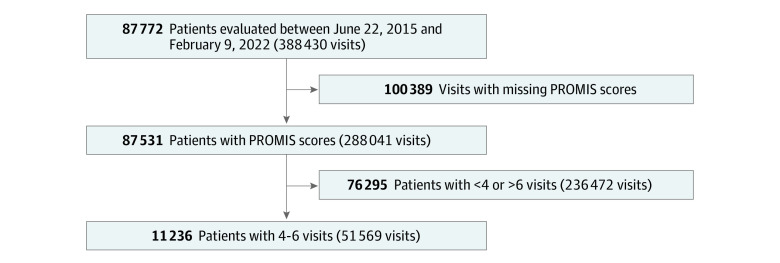
Inclusion Flowchart PROMIS indicates Patient-Reported Outcomes Measurement Information System.

Of 87 772 patients who were evaluated at the study institution during the study period, 11 236 were eligible for inclusion in the primary cohort (51 569 total visits) (Figure 1). This cohort had a mean (SD) age of 58 (16) years; 1288 (12%) were Black, 9706 (86%) were White, and 7218 (64%) were women. At baseline, the cohort reported similar symptoms of anxiety (mean [SD] PROMIS Anxiety score, 48.2 [9.1]) and depression (PROMIS Depression score, 46.0 [8.6]) compared with the general US population. Patients who achieved meaningfully improved physical function (1672 patients) and/or pain interference (1391 patients) during the study period reported similar baseline symptoms of anxiety and depression than those who did not achieve meaningfully improved physical function and/or pain interference (Table 1).

**Table 1.** Patient Characteristics

Characteristics	Patients, No. (%)		
	Primary cohort (N = 11 236)	Physical function improved (n = 1672)^a^	Pain interference improved (n = 1391)^a^
Age, mean (SD), y	58 (16)	57 (16)	58 (15)
Gender			
Men	4018 (36)	605 (36)	463 (33)
Women	7218 (64)	1067 (64)	928 (67)
Race			
American Indian or Alaska Native	26 (<1)	1 (<1)	2 (<1)
Asian	120 (1)	15 (<1)	15 (1)
Black or African American	1288 (12)	190 (11)	161 (12)
White	9706 (86)	1455 (87)	1203 (87)
Other Pacific Islander	15 (<1)	2 (<1)	1 (<1)
Multiracial	39 (<1)	5 (<1)	1 (<1)
Unable to answer	1 (<1)	0	1 (<1)
Declined	41 (<1)	4 (<1)	7 (<1)
Baseline PROMIS scores, mean (SD)^b^			
Anxiety	48.2 (9.1)	49.3 (9.3)	50.7 (9.2)
Depression	46.0 (8.6)	46.8 (8.9)	47.9 (9.0)
Physical Function	40.7 (8.5)	33.8 (7.4)	37.0 (7.8)
Pain Interference	58.8 (7.8)	62.2 (7.7)	65.3 (6.4)
Final PROMIS scores, mean (SD)^b^			
Anxiety	60.6 (9.0)	58.3 (9.1)	56.3 (9.8)
Depression	54.2 (9.3)	52.1 (9.2)	51.3 (9.0)
Physical Function	34.7 (8.1)	43.0 (7.8)	41.2 (8.1)
Pain Interference	65.3 (7.6)	60.3 (8.0)	55.3 (7.5)
Time from baseline to final follow-up, mean (SD), d	419 (192)	374 (170)	603 (151)

^a^ Improved was defined as a 5-point favorable change in patients’ Patient-Reported Outcomes Measurement Information System (PROMIS) scores from the first to last clinic visit during the study period (ie, 5-point score increase in PROMIS Physical Function, 5-point score decrease in PROMIS Pain Interference).

^b^ Baseline and final mean PROMIS scores for each PROMIS domain were statistically significantly different between each of the improved cohorts (ie, “physical function improved” and “pain interference improved”) and the remaining patients who did not meet the improved criteria (all *P* < .001).

### Associations With Anxiety

**Figure 2.**
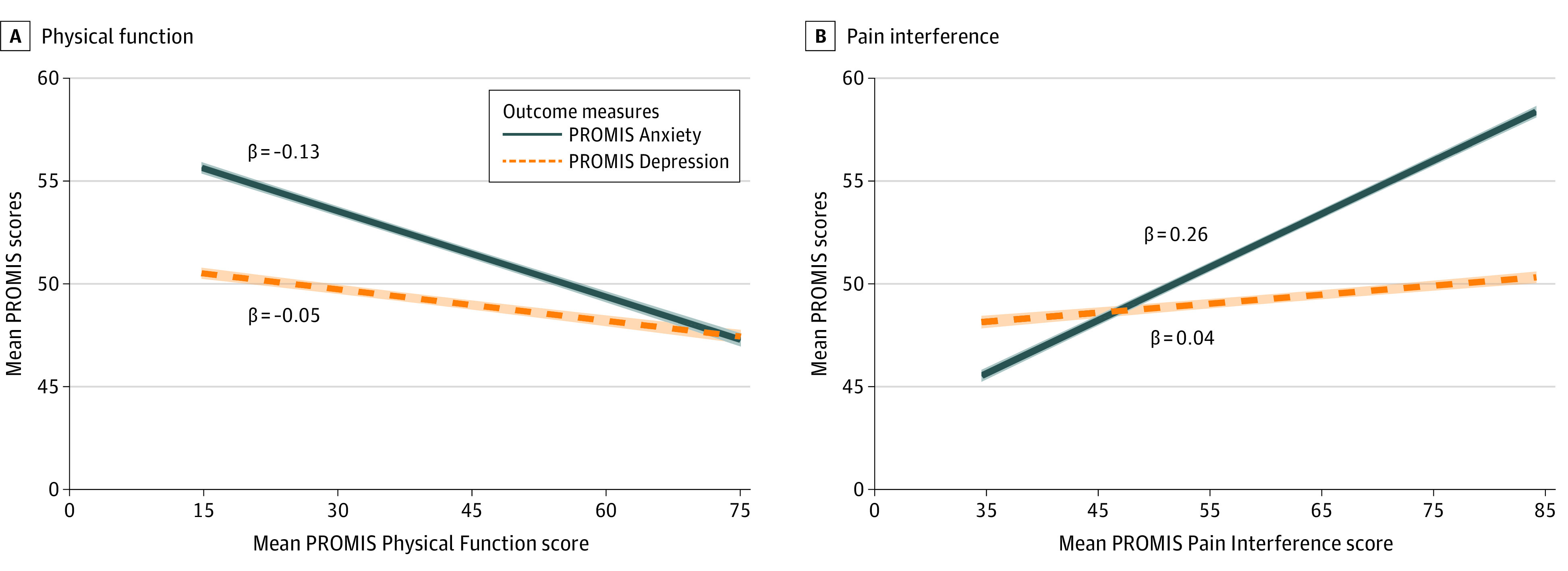
Projected Patient-Reported Outcomes Measurement Information System (PROMIS) Mental Health Scores as a Function of PROMIS Physical Health Scores Projected scores were adjusted for age, gender, race, and shared variance between PROMIS Depression and Anxiety. Shading indicates 95% CIs.

For the primary cohort after adjusting for age, gender, race, and depression symptoms, improvements in physical function (β = −0.14; 95% CI, −0.15 to −0.13; *P* for FDR < .001) and pain interference (β = 0.26; 95% CI, 0.25 to 0.26; *P* for FDR < .001) were each associated with statistically and meaningfully improved anxiety symptoms (Figure 2; Table 2). To reach a clinically meaningful improvement in anxiety symptoms of at least 3.0 PROMIS Anxiety points, an associated improvement of 21 or more (95% CI, 20 to 23) PROMIS points on Physical Function or 12 or more (95% CI, 12 to 12) points on Pain Interference than would be expected (calculated as 3.0/β).

**Table 2.** Models Testing for Associations Between PROMIS Physical Function and Pain Interference With PROMIS Anxiety

Variable	Physical function model		Pain interference model	
	β (95% CI)	*P *value	β (95% CI)	*P *value
Intercept	29.83 (29.24 to 30.42)	<.001	10.45 (9.89 to 11.01)	<.001
PROMIS Physical Function	−0.14 (−0.15 to −0.13)	<.001	NI	NI
PROMIS Pain Interference	NI	NI	0.26 (0.25 to 0.26)	<.001
PROMIS Depression	0.58 (0.58 to 0.59)	<.001	0.54 (0.54 to 0.55)	<.001
Age (per year)	−0.02 (−0.02 to −0.01)	<.001	−0.01 (−0.02 to −0.01)	<.001
Gender (man)	−0.03 (−0.20 to 0.13)	.70	−0.12 (−0.28 to 0.03)	.13
Race (White)	−2.23 (−2.46 to −2.00)	<.001	−1.79 (−2.01 to −1.57)	<.001
Visit 2	2.44 (2.32 to 2.57)	<.001	2.29 (2.16 to 2.41)	<.001
Visit 3	3.92 (3.79 to 4.05)	<.001	3.65 (3.53 to 3.78)	<.001
Visit 4	5.68 (5.55 to 5.81)	<.001	5.28 (5.15 to 5.41)	<.001
Visit 5	6.50 (6.33 to 6.67)	<.001	6.05 (5.88 to 6.22)	<.001
Visit 6	7.59 (7.32 to 7.85)	<.001	7.01 (6.75 to 7.27)	<.001
Random effects				
σ^2^	22.95	NA	21.46	NA
τ_00,id_	12.76	NA	12.15	NA
ICC	0.36	NA	0.36	NA
Total patients, No.	11 236	NA	11 236	NA
Observations, No.	51 569	NA	51 569	NA
Marginal *R^2^*/conditional *R^2^*^a^	0.561/0.718	NA	0.585/0.735	NA

Abbreviations: ICC, intraclass correlation coefficient; NA, not applicable; NI, not included in the model; PROMIS, Patient-Reported Outcomes Measurement Information System; σ^2^, random effect variance; τ_00,id_, random intercept for each individual patient.

^a^ *R^2^* indicates the total variance in the data that is explained by fixed effects alone (marginal *R^2^*) and by fixed and random effects together (conditional *R^2^*).

Among the subgroup of patients who achieved meaningfully improved physical function or pain interference during the study period (of at least 5 PROMIS points), these associations were similar (physical function: β = −0.11; 95% CI, −0.13 to −0.09; *P* for FDR < .001; pain interference: β = 0.22; 95% CI, 0.19 to 0.24; *P* for FDR < .001) (eTable 1 in Supplement 1). However, compared with the primary cohort, a somewhat greater associated improvement of 27 or more (95% CI, 23 to 33) PROMIS points on Physical Function or 14 or more (95% CI, 13 to 16) points on Pain Interference would be expected in patients who achieve a clinically meaningful improvement in anxiety symptoms of at least 3.0 PROMIS points.

### Associations With Depression

For the primary cohort after adjusting for age, gender, race, and anxiety symptoms, improvements in physical function (β = −0.05; 95% CI, −0.06 to −0.04; *P* for FDR < .001) and pain interference (β = 0.04; 95% CI, 0.04 to 0.05; *P* for FDR < .001) were each associated with statistically but not meaningfully improved depression symptoms (Figure 2, Table 3). That is, to reach a clinically meaningful improvement in depression symptoms of at least 3.2 PROMIS Depression points, an associated improvement of 64 or more (95% CI, 53 to 80) PROMIS points on Physical Function or 64 or more (95% CI, 64 to 80) points on Pain Interference would be expected (calculated as 3.2/β), which was not possible based on the actual score ranges of these PROMIS measures.

**Table 3.** Models Testing for Associations Between PROMIS Physical Function and Pain Interference With PROMIS Depression

Variable	Physical function model		Pain interference model	
	β (95% CI)	*P *value	β (95% CI)	*P *value
Intercept	17.34 (16.71 to 17.96)	<.001	12.65 (12.08 to 13.22)	<.001
PROMIS Physical Function	−0.05 (−0.06 to −0.04)	<.001	NI	NI
PROMIS Pain Interference	NI	NI	0.04 (0.04 to 0.05)	<.001
PROMIS Anxiety	0.64 (0.64 to 0.65)	<.001	0.64 (0.63 to 0.65)	<.001
Age (per year)	−0.01 (−0.02 to −0.01)	<.001	−0.01 (−0.01 to −0.01)	<.001
Gender (man)	−0.17 (−0.32 to −0.01)	.03	−0.21 (−0.36 to −0.06)	.006
Race (White)	0.61 (0.30 to 0.73)	<.001	0.66 (0.48 to 0.88)	<.001
Visit 2	−0.20 (−0.33 to −0.06)	.005	−0.18 (−0.32 to −0.04)	.01
Visit 3	−0.29 (−0.43 to −0.15)	<.001	−0.27 (−0.41 to −0.13)	<.001
Visit 4	−0.46 (−0.61 to −0.31)	<.001	−0.44 (−0.59 to −0.29)	<.001
Visit 5	−0.45 (−0.64 to 0.26)	<.001	−0.42 (−0.61 to −0.23)	<.001
Visit 6	−0.26 (−0.55 to −0.03)	.08	−0.24 (−0.53 to −0.06)	.11
Random effects				
σ^2^	26.39	NA	26.40	NA
τ_00,id_	9.38	NA	9.51	NA
ICC	0.26	NA	0.26	NA
Total patients, No.	11 236	NA	11 236	NA
Observations, No.	51 569	NA	51 569	NA
Marginal *R^2^*/conditional *R^2^*^a^	0.514/0.641	NA	0.511/0.641	NA

Abbreviations: ICC, intraclass correlation coefficient; NA, not applicable; NI, not included in the model; PROMIS, Patient-Reported Outcomes Measurement Information System; σ^2^, random effect variance; τ_00,id_, random intercept for each individual patient.

^a^ *R^2^* indicates the total variance in the data that is explained by fixed effects alone (marginal *R^2^*) and by fixed and random effects together (conditional *R^2^*).

Among the subgroup of patients who achieved meaningfully improved physical function or pain interference during the study period (of at least 5 PROMIS points), these associations were unchanged (physical function: β = −0.03; 95% CI, −0.05 to −0.02; *P* for FDR < .001; pain interference: β = 0.04; 95% CI, 0.02 to 0.06; *P* for FDR < .001) (eTable 2 in Supplement 1). Specifically, the models for this subgroup still suggest that it would not be possible to reach a clinically meaningful improvement in depression symptoms of 3.2 PROMIS points due only to associated improvements in physical function or pain interference.

### Generalizability Analysis

Compared with our primary cohort of patients who had at least 4 clinic visits during the study period, no meaningful differences in baseline characteristics (eTable 3 in Supplement 1) or clinically significant associations of physical with mental health were observed in our generalizability cohort of patients who only had 3 visits during the 6-year study period. eResults, eTable 4, and eTable 5 in Supplement 1 present these findings.

## Discussion

In this large cohort of patients who sought care for 1 or more musculoskeletal conditions, large improvements in physical function and pain interference were associated with modestly improved anxiety symptoms but were not associated with any meaningful improvement in depression symptoms. These study findings were consistent, even when considering: (1) all patients vs only patients who achieved meaningfully improved physical function or pain interference during the study period, and (2) patients with 4 to 6 clinic visits vs only 3 visits during the study period.

There is strong evidence that (1) preexisting anxiety and depression symptoms worsen physical health–related outcomes after musculoskeletal treatment, and (2) improving anxiety and depression symptoms can contribute to improved physical function and reduced pain.^10,14,15,16,17,18,19,47,48,49^ However, our results suggest that the converse is not true. That is, improved physical health is not necessarily associated with meaningfully improved anxiety or depression symptoms. Other emerging research regarding the association between improved physical health and subsequent symptoms of anxiety and depression has been mixed.^20,21,22^ For example, a cohort of young adults reported a decrease in utilization of mental health treatment after they underwent hip surgery,^21^ but in contrast, symptoms of depression did not meaningfully change after a different cohort of somewhat older patients underwent orthopedic treatment for a hand condition.^22^ It is possible that some musculoskeletal treatments may improve symptoms of anxiety and depression in the short-term for patients who, for instance, may have situational or state (rather than trait) anxiety or depression.^50^ This could especially be true for elective orthopedic procedures which have a high success rate and are typically only performed on people who are generally healthy at baseline.

In contrast to previous studies, this study was not designed to assess the effectiveness of a specific musculoskeletal treatment for a specific patient population. Rather, this study was designed to identify broad associations between physical and mental health trajectories over a long time period, regardless of a patient’s precise musculoskeletal condition(s) or structure-based treatment (eg, surgery, injection, physical therapy, etc). Our findings suggest that over the long-term, musculoskeletal clinicians should be aware that improvements in physical function and/or pain interference are not necessarily associated with meaningful and sustained improvements in symptoms of anxiety or especially depression. Furthermore, because preexisting symptoms of anxiety and depression are associated with worse orthopedic outcomes, we advocate for musculoskeletal clinicians to be equipped with the training and referral resources to address mental health as part of patient counseling and the musculoskeletal treatment plan.^28^

### Limitations

Although this study had key strengths including the large sample size and relatively long follow-up duration of over 6 years, there were also several limitations. First, this was an observational study, which limits the causality we can attribute to the associations between physical and mental health that we identified. Second, the patient cohort had limited racial diversity. Third, additional sociodemographic and clinical variables were not available to be included as possible confounders (eg, financial considerations, social support, ethnicity, clinical diagnosis of anxiety and/or depression, musculoskeletal and mental health related interventions, and medical comorbidities).^20^ It is also possible that other patient, diagnosis, and treatment characteristics could affect the associations we identified (eg, traumatic vs degenerative conditions, spine vs peripheral joint conditions, definitive vs palliative treatment intent, etc).

## Conclusions

This large cohort study suggests that over the course of several years, improvements in physical function and pain interference may be associated with improvement in symptoms of anxiety but not of depression. Furthermore, substantial improvements in physical function and pain interference are expected for patients to reach clinically meaningful associations with improvement in anxiety symptoms. Therefore, musculoskeletal clinicians and patients cannot assume that exclusively structure-based treatment of a musculoskeletal condition will necessarily result in improved symptoms of depression or potentially even anxiety. We advocate for clinicians to thoughtfully and intentionally address the mental health–related contributors to, and sequelae of, musculoskeletal conditions when counseling patients and creating person-centered treatment plans. Further investigation is needed to identify methods of addressing mental health in the context of musculoskeletal care that are both feasible and effective.

## Notes

Supplement 1.
eMethods.

eResults.
**eFigure.** PROMIS Physical Health Score Changes Among Patients Who Achieved Meaningful Improvement **eTable 1.** Main Effects of PROMIS Physical Function and Pain Interference on PROMIS Anxiety Among Patients With Clinically Improved Physical Function and Pain Interference, Respectively **eTable 2.** Main Effects of PROMIS Physical Function and Pain Interference on PROMIS Depression Among Patients With Clinically Improved Physical Function and Pain Interference, Respectively **eTable 3.** Patient Characteristics of the Generalizability Cohort Who Had Three Clinic Visits During the Study Period **eTable 4.** Main Effects of PROMIS Physical Function and Pain Interference on PROMIS Anxiety in the Generalizability Cohort **eTable 5.** Main Effects of PROMIS Physical Function and Pain Interference on PROMIS Depression in the Generalizability Cohort

Supplement 2.
Data Sharing Statement
