# Supplementary material for: Improvements in Physical Function and Pain Interference and Changes in Mental Health Among Patients Seeking Musculoskeletal Care
Source: JAMA Netw Open. 2023 Jun 28;6(6):e2320520. doi: 10.1001/jamanetworkopen.2023.20520 (PMC10308248; doi:10.1001/jamanetworkopen.2023.20520)
Supplement: Supplement 1. — eMethods. eResults. eFigure. PROMIS Physical Health Score Changes Among Patients Who Achieved Meaningful Improvement eTable 1. Main Effects of PROMIS Physical Function and Pain Interference on PROMIS Anxiety Among Patients With Clinically Improved Physical Function and Pain Interference, Respectively eTable 2. Main Effects of PROMIS Physical Function and Pain Interference on PROMIS Depression Among Patients With Clinically Improved Physical Function and Pain Interference, Respectively eTable 3. Patient Characteristics of the Generalizability Cohort Who Had Three Clinic Visits During the Study Period eTable 4. Main Effects of PROMIS Physical Function and Pain Interference on PROMIS Anxiety in the Generalizability Cohort eTable 5. Main Effects of PROMIS Physical Function and Pain Interference on PROMIS Depression in the Generalizability Cohort [file jamanetwopen-e2320520-s001.pdf]

Supplemental Online Content

Zhang W, Singh SP, Clement A, Calfee RP, Bijsterbosch JD, Cheng AL. Improvements in physical function and pain interference and changes mental health among patients seeking musculoskeletal care. *JAMA Netw Open.* 2023;6(6):e2320520. doi:10.1001/jamanetworkopen.2023.20520

eMethods.

eResults.

eFigure. PROMIS Physical Health Score Changes Among Patients Who Achieved Meaningful Improvement

eTable 1. Main Effects of PROMIS Physical Function and Pain Interference on PROMIS Anxiety Among Patients With Clinically Improved Physical Function and Pain Interference, Respectively

eTable 2. Main Effects of PROMIS Physical Function and Pain Interference on PROMIS Depression Among Patients With Clinically Improved Physical Function and Pain Interference, Respectively

eTable 3. Patient Characteristics of the Generalizability Cohort Who Had Three Clinic Visits During the Study Period

eTable 4. Main Effects of PROMIS Physical Function and Pain Interference on PROMIS Anxiety in the Generalizability Cohort

eTable 5. Main Effects of PROMIS Physical Function and Pain Interference on PROMIS Depression in the Generalizability Cohort

This supplemental material has been provided by the authors to give readers additional information about their work.

© 2023 Zhang W et al. *JAMA Network Open.*

eMethods

Eligibility criteria.

Eligibility criteria related to patients' number of clinic visits during the study period was guided by the goals of: 1) including maximal data points (i.e., maximal number of visits) per patient in order to optimally estimate longitudinal trajectories, and 2) maintaining sufficient statistical power per visit. We used a "rule-of-thumb" calculation to allow a minimum of 20 observations/patients per parameter in our linear mixed effects models, and we found that a sufficient number of patients had at least four clinic visits during the study period (N=13,874). We then excluded patients with more than six clinic visits during the study period because the sample size of those patients would be potentially underpowered (i.e., N=3,543 for six visits, N=1,748 for seven visits).

Linear Mixed Effects Model (LMM) Specifications.

In the current study, we ran the following linear mixed effects models using PROMIS measures to operationalize Anxiety, Depression, Physical Function, and Pain Interference:

1. **Anxiety** ~ <u>Physical Function</u> + *Depression* + *Age* + *Gender* + *Race* + *Visit* + (1|Patient) + (1|Visit)
2. **Depression** ~ <u>Physical Function</u> + *Anxiety* + *Age* + *Gender* + *Race* + *Visit* + (1|Patient) + (1|Visit)
3. **Anxiety** ~ <u>Pain Interference</u> + *Depression* + *Age* + *Gender* + *Race* + *Visit* + (1|Patient) + (1|Visit)
4. **Depression** ~ <u>Pain Interference</u> + *Anxiety* + *Age* + *Gender* + *Race* + *Visit* + (1|Patient) + (1|Visit)

This model specification allowed us to examine the hypothesized associations between outcome measures (in bold) and exposure measures (underlined), while also accounting for: 1) the shared variance between anxiety and depression, 2) confounding variables (in italic), and 3) the random effects of individual patients and of each clinic visit (in parentheses).

Note, "Visit" is a factorial variable with six levels for the primary cohort and three levels for the generalizability cohort, which accounts for the non-independent data structure associated with this variable. For example, assessment of anxiety and depression for a particular patient at one specific visit is not independent from the assessments of this patient at other clinic visits, and different patients at the same visit level (i.e., number) may have been influenced by similar unmeasured confounding variables (e.g., similar recovery stage). For all models, we initially included a random intercept for each "Patient" to account for individual-level variability (e.g., different between-visit time intervals across patients) and a random intercept for each clinic "Visit" to account for variability across visits (e.g., varying injury characteristics associated with the total number of clinic visits during the study period). Whenever models failed to converge after maximizing the number of iterations, we dropped the random intercept estimation for "visit" to facilitate model convergence. All resulting statistical models converged.

© 2023 Zhang W et al. *JAMA Network Open.*

eResults

Associations with anxiety.

For the generalizability cohort of patients who had only three visits during the six-year study period, after adjusting for age, gender, race, and depression symptoms, improvements in physical function (β=-0.16 [95% CI -0.16 to -0.15], $p_{fdr}<0.001$) and pain interference (β=0.27 [0.26 to 0.28], $p_{fdr}<0.001$) were each associated with statistically and meaningfully improved anxiety symptoms (eTable 4). To reach a clinically meaningful improvement in anxiety symptoms of at least 3.0 PROMIS Anxiety points, an associated improvement of ≥19 [19 to 20] PROMIS points on Physical Function or ≥11 [11 to 12] points on Pain Interference would be expected (calculated as 3.0/β).

Associations with depression.

For the generalizability cohort of patients who had only three visits during the six-year study period, after adjusting for age, gender, race, and anxiety symptoms, improvements in physical function (β=-0.04 [-0.05 to -0.03], $p_{fdr}<0.001$) and pain interference (β=0.03 [0.02 to 0.04], $p_{fdr}<0.001$) were associated with statistically but not meaningfully improved depression symptoms (eTable 5). That is, to reach a clinically meaningful improvement in depression symptoms of at least 3.2 PROMIS Depression points, an associated improvement of ≥80 [64 to 107] PROMIS points on Physical Function or ≥107 [80 to 160] points on Pain Interference would be expected (calculated as 3.2/β), which is not possible based on the actual score ranges of these PROMIS measures.

© 2023 Zhang W et al. *JAMA Network Open.*

eFigure. PROMIS physical health score changes among patients who achieved meaningful improvement.

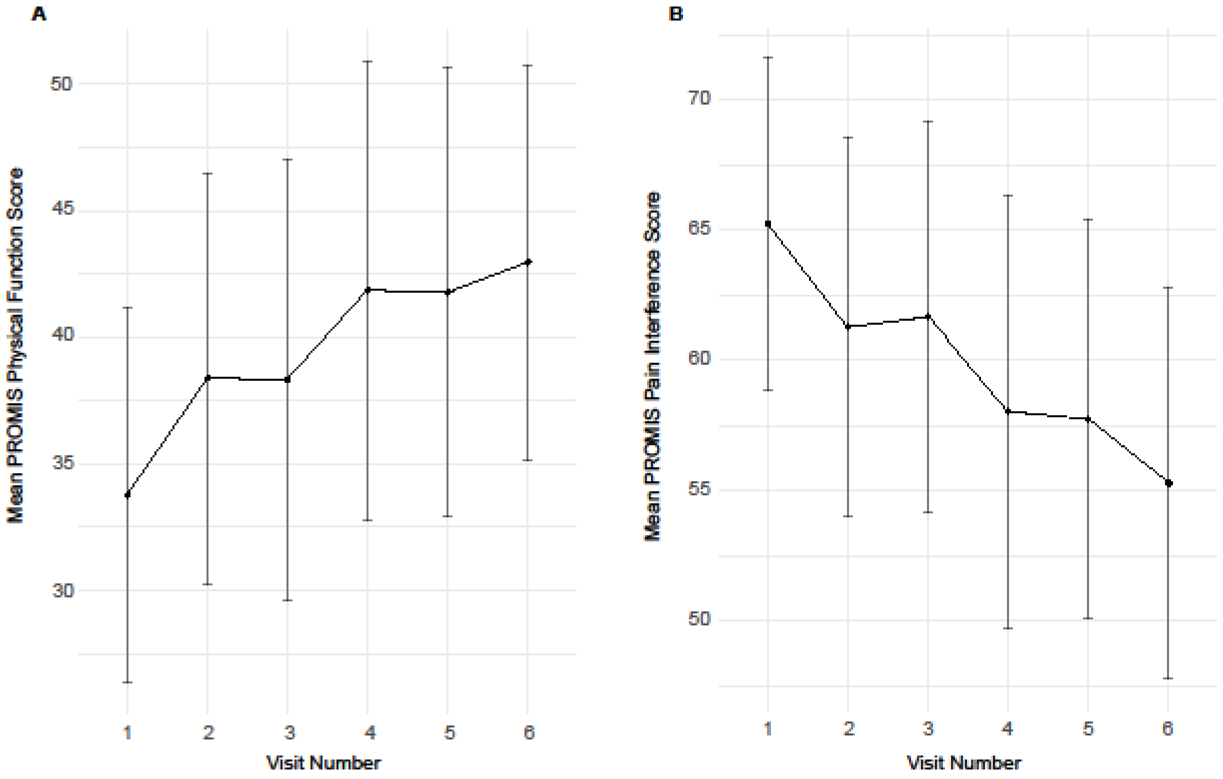

Mean PROMIS (A) Physical Function (N=1,672) and (B) Pain Interference (N=1,391) scores over time among patients who achieved meaningfully improved physical function or pain interference (of at least five PROMIS points) between their first and last clinic visit during the study period. Error bars represent one standard deviation.
Abbreviation: PROMIS (Patient-Reported Outcomes Measurement Information System).

© 2023 Zhang W et al. *JAMA Network Open.*

eTable 1: Main effects of PROMIS Physical Function and Pain Interference on PROMIS Anxiety, among patients with clinically improved[a] physical function and pain interference, respectively.

	Physical Function Model		Pain Interference Model	
**Predictor**	**β (95% CI)**	***p***	**β (95% CI)**	***p***
(Intercept)	25.82 (24.32 to 27.32)	<0.001	9.11 (6.04 to 12.18)	<0.001
PROMIS Physical Function	-0.11 (-0.13 to -0.09)	<0.001	NI	NI
PROMIS Pain Interference	NI	NI	0.22 (0.19 to 0.24)	<0.001
PROMIS Depression	0.65 (0.63 to 0.66)	<0.001	0.63 (0.61 to 0.65)	<0.001
Age (per year)	-0.02 (-0.03 to -0.01)	0.005	-0.02 (-0.03 to 0.001)	0.008
Gender (Man)	0.17 (-0.26 to 0.61)	0.43	-0.20 (-0.66 to 0.26)	0.39
Race (White)	-2.38 (-3.00 to -1.76)	<0.001	-1.50 (-2.13 to -0.86)	<0.001
Visit 2	2.68 (2.32 to 3.04)	<0.001	2.30 (-1.25 to 5.84)	0.20
Visit 3	3.58 (3.22 to 3.94)	<0.001	2.91 (-0.63 to 6.46)	0.11
Visit 4	5.14 (4.76 to 5.52)	<0.001	4.60 (1.05 to 8.14)	0.01
Visit 5	6.12 (5.62 to 6.62)	<0.001	5.59 (2.02 to 9.15)	0.002
Visit 6	7.34 (6.57 to 8.12)	<0.001	6.44 (2.82 to 10.06)	<0.001
**Random Effects**				
σ^2	25.80	-	25.62	-
$\tau_{00,id}$	12.93	-	11.06	-
$\tau_{00,visit}$	NI	-	1.61	
ICC	0.33	-	0.33	-
N_{id}	1,672	-	1,391	-
N_{visit}	NI	-	6	
Observations	7,652	-	6,366	-
[b]Marginal R^2 / Conditional R^2	0.532 / 0.688	-	0.524 / 0.681	-

[a]"Clinically improved" physical function or pain interference was defined as a five-point favorable change in patients' Patient-Reported Outcomes Measurement Information System (PROMIS) scores from the first to last clinic visit during the study period (i.e., five-point score increase in PROMIS Physical Function, five-point score decrease in PROMIS Pain Interference).
[b]R^2 indicates the total variance in the data that is explained by fixed effects alone (Marginal R^2) and by fixed and random effects together (Conditional R^2).
Abbreviations: PROMIS (Patient-Reported Outcomes Measurement Information System), NI (Not included in the model), σ^2 (random effect variance), $\tau_{00,id}$ (random intercept for each individual patient), $\tau_{00,visit}$ (random intercept for each clinic visit), ICC (Intraclass correlation coefficient), N_{id} (number of patients), N_{visit} (number of clinic visits), Observations (number of datapoints for all included patients across all visits).

© 2023 Zhang W et al. *JAMA Network Open.*

eTable 2: Main effects of PROMIS Physical Function and Pain Interference on PROMIS Depression, among patients with clinically improved[a] physical function and pain interference, respectively.

	Physical Function Model		Pain Interference Model	
**Predictor**	**β (95% CI)**	***p***	**β (95% CI)**	***p***
(Intercept)	16.13 (14.60 to 17.65)	<0.001	13.10 (9.71 to 16.49)	<0.001
PROMIS Physical Function	-0.03 (-0.05 to -0.02)	<0.001	NI	NI
PROMIS Pain Interference	NI	NI	0.04 (0.02 to 0.06)	<0.001
PROMIS Anxiety	0.65 (0.63 to 0.67)	<0.001	0.64 (0.62 to 0.66)	<0.001
Age (per year)	0.00 (-0.01 to 0.01)	0.70	-0.01 (-0.02 to 0.01)	0.34
Gender (Man)	-0.56 (-0.97 to -0.16)	0.005	-0.04 (-0.49 to 0.42)	0.80
Race (White)	0.26 (-0.32 to 0.84)	0.53	0.29 (-0.35 to 0.93)	0.51
Visit 2	-0.18 (-0.55 to 0.18)	0.33	-0.09 (-4.18 to 4.01)	0.97
Visit 3	-0.07 (-0.45 to 0.30)	0.70	-0.08 (-4.17 to 4.01)	0.97
Visit 4	-0.19 (-0.60 to 0.21)	0.35	-0.10 (-4.19 to 4.00)	0.96
Visit 5	-0.43 (-0.95 to 0.09)	0.11	-0.44 (-4.55 to 3.67)	0.83
Visit 6	-0.82 (-1.62 to -0.02)	0.05	-0.38 (-4.54 to 3.78)	0.86
**Random Effects**				
σ^2	26.63	-	26.14	-
$\tau_{00,id}$	10.42	-	10.92	-
$\tau_{00,visit}$	NI	-	2.16	
ICC	0.28	-	0.33	-
N_{id}	1,672	-	1,391	-
N_{visit}	NI	-	6	
Observations	7,625	-	6,366	-
[b]Marginal R^2 / Conditional R^2	0.502 / 0.642	-	0.468 / 0.646	-

[a]"Clinically improved" physical function or pain interference was defined as a five-point favorable change in patients' Patient-Reported Outcomes Measurement Information System (PROMIS) scores from the first to last clinic visit during the study period (i.e., five-point score increase in PROMIS Physical Function, five-point score decrease in PROMIS Pain Interference).
[b]R^2 indicates the total variance in the data that is explained by fixed effects alone (Marginal R^2) and by fixed and random effects together (Conditional R^2).
Abbreviations: PROMIS (Patient-Reported Outcomes Measurement Information System), NI (Not included in the model), σ^2 (random effect variance), $\tau_{00,id}$ (random intercept for each individual patient), $\tau_{00,visit}$ (random intercept for each clinic visit), ICC (Intraclass correlation coefficient), N_{id} (number of patients), N_{visit} (number of clinic visits), Observations (number of datapoints for all included patients across all visits).

© 2023 Zhang W et al. *JAMA Network Open.*

eTable 3: Patient characteristics of the generalizability cohort who had three clinic visits during the study period.

	Generalizability cohort (N=11,501)
Age, Mean (SD)	56 (16)
Gender, N (%)	
Men	4,535 (39)
Women	6,966 (61)
Race, N (%)	
American Indian or Alaska Native	18 (< 1)
Asian	171 (2)
Black or African American	1,342 (12)
White	9,864 (86)
Other Pacific Islander	12 (< 1)
Multi-racial	47 (< 1)
Unable to answer	5 (< 1)
Declined	42 (< 1)
Baseline PROMIS scores, Mean (SD)	
Anxiety	48.4 (9.4)
Depression	45.5 (8.9)
Physical Function	41.2 (8.9)
Pain Interference	58.8 (8.0)
Final PROMIS scores, Mean (SD)	
Anxiety	57.7 (8.6)
Depression	51.1 (9.0)
Physical Function	37.8 (8.6)
Pain Interference	63.1 (7.4)
Days from baseline to final follow-up, Mean (SD)	283 (168)

Abbreviation: PROMIS (Patient-Reported Outcomes Measurement Information System).

© 2023 Zhang W et al. *JAMA Network Open.*

eTable 4: Main effects of PROMIS Physical Function and Pain Interference on PROMIS Anxiety in the generalizability cohort.

	Physical Function Model		Pain Interference Model	
**Predictor**	**β (95% CI)**	***p***	**β (95% CI)**	***p***
(Intercept)	31.43 (23.86 to 39.0)	<0.001	10.20 (3.14 to 17.27)	<0.001
PROMIS Physical Function	-0.16 (-0.16 to -0.15)	<0.001	NI	NI
PROMIS Pain Interference	NI	NI	0.27 (0.26 to 0.28)	<0.001
PROMIS Depression	0.58 (0.58 to 0.59)	<0.001	0.55 (0.54 to 0.56)	<0.001
Age (per year)	-0.02 (-0.03 to -0.02)	<0.001	-0.02 (-0.02 to -0.01)	<0.001
Gender (Man)	-0.38 (-0.55 to -0.20)	<0.001	-0.52 (-0.68 to -0.35)	0.02
Race (White)	-2.16 (-2.40 to -1.92)	<0.001	-1.72 (-1.95 to -1.48)	<0.001
Visit 2	2.90 (-7.76 to 13.56)	0.59	2.66 (-7.29 to 16.21)	0.60
Visit 3	5.57 (-5.09 to 16.23)	0.31	5.09 (-4.86 to 15.05)	0.32
**Random Effects**				
σ^2	22.58	-	21.27	-
$\tau_{00,id}$	13.82	-	12.86	-
$\tau_{00,visit}$	14.78		12.89	
ICC	0.56	-	0.55	-
N_{id}	11,501	-	11,501	-
N_{visit}	3	-	3	-
Observations	34,503	-	34,503	-
[a]Marginal R^2 / Conditional R^2	0.487 / 0.774	-	0.518 / 0.782	-

[a]R^2 indicates the total variance in the data that is explained by fixed effects alone (Marginal R^2) and by fixed and random effects together (Conditional R^2).

Abbreviations: PROMIS (Patient-Reported Outcomes Measurement Information System), NI (Not included in the model), σ^2 (random effect variance), $\tau_{00,id}$ (random intercept for each individual patient), $\tau_{00,visit}$ (random intercept for each clinic visit), ICC (Intraclass correlation coefficient), N_{id} (number of patients), N_{visit} (number of clinic visits), Observations (number of datapoints for all included patients across all visits).

© 2023 Zhang W et al. *JAMA Network Open.*

eTable 5: Main effects of PROMIS Physical Function and Pain Interference on PROMIS Depression in the generalizability cohort.

	Physical Function Model		Pain Interference Model	
**Predictor**	**β (95% CI)**	***p***	**β (95% CI)**	***p***
(Intercept)	14.53 (12.73 to 16.33)	0.007	11.13 (9.83 to 12.43)	<0.001
PROMIS Physical Function	-0.04 (-0.05 to -0.03)	<0.001	NI	NI
PROMIS Pain Interference	NI	NI	0.03 (0.02 to 0.04)	<0.001
PROMIS Anxiety	0.67 (0.66 to 0.68)	<0.001	0.67 (0.66 to 0.68)	<0.001
Age (per year)	0.00 (-0.01 to 0.00)	0.29	0.00 (-0.01 to 0.00)	0.86
Gender (Man)	-0.03 (-0.20 to 0.14)	0.74	-0.07 (-0.24 to 0.10)	0.41
Race (White)	0.49 (0.25 to 0.72)	<0.001	0.51 (0.27 to 0.75)	<0.001
Visit 2	-0.16 (-2.48 to 2.16)	0.89	-0.16 (-1.74 to 1.42)	0.84
Visit 3	-0.66 (-2.98 to 1.66)	0.58	-0.67 (-2.25 to 0.92)	0.41
**Random Effects**				
σ^2	27.55	-	27.57	-
$\tau_{00,id}$	10.82	-	10.88	-
$\tau_{00,visit}$	0.70		0.32	
ICC	0.29	-	0.29	-
N_{id}	11,501	-	11,501	-
N_{visit}	3	-	3	
Observations	34,503	-	34,503	-
[a]Marginal R^2 / Conditional R^2	0.514 / 0.657	-	0.515 / 0.655	-

[a]R^2 indicates the total variance in the data that is explained by fixed effects alone (Marginal R^2) and by fixed and random effects together (Conditional R^2).

Abbreviations: PROMIS (Patient-Reported Outcomes Measurement Information System), NI (Not included in the model), σ^2 (random effect variance), $\tau_{00,id}$ (random intercept for each individual patient), $\tau_{00,visit}$ (random intercept for each clinic visit), ICC (Intraclass correlation coefficient), N_{id} (number of patients), N_{visit} (number of clinic visits), Observations (number of datapoints for all included patients across all visits).

© 2023 Zhang W et al. *JAMA Network Open.*
