# Supplementary material for: Improvements in Physical Function and Pain Interference and Changes in Mental Health Among Patients Seeking Musculoskeletal Care
Source: JAMA Netw Open. 2023 Jun 28;6(6):e2320520. doi: 10.1001/jamanetworkopen.2023.20520 (PMC10308248; doi:10.1001/jamanetworkopen.2023.20520)
Supplement: Supplement 2. — Data Sharing Statement [file jamanetwopen-e2320520-s002.pdf]

Data Sharing Statement

Zhang. Improvements in Physical Function and Pain Interference and Changes in Mental Health Among Patients Seeking Musculoskeletal Care. *JAMA Netw Open*. Published June 28, 2023. doi:10.1001/jamanetworkopen.2023.20520

Data
Data available: Yes
Data types: Deidentified participant data
How to access data: All de-identified data produced in the present study are available upon reasonable request to the authors (chengal@wustl.edu).
When available: With publication

Supporting Documents
Document types: None

Additional Information
Who can access the data: Researchers whose proposed use of the data has been approved.
Types of analyses: For any purpose.
Mechanisms of data availability: With investigator support, after approval of a proposal and with a signed data access agreement.
